# Viral Genomics and Bioinformatics

**DOI:** 10.3390/v2122587

**Published:** 2010-11-30

**Authors:** Donald Seto

**Affiliations:** Department of Bioinformatics and Computational Biology, George Mason University, Manassas, VA 20110, USA; E-Mail: dseto@gmu.edu; Tel.: +1-703-993-8403; Fax: +1-703-993-8401

## 1. Introduction

From the recognition by Ivanovski in 1892 that tobacco mosaic disease is caused and transmitted by fine pore filtrates [1], viruses have been isolated, characterized, identified and studied from animals, plants, protists, bacteria and even other viruses [2,3]. As human and global public health pathogens that can be highly contagious and have devastating morbidity and mortality consequences, viruses are the focus of much research. The difficult challenge has been to define and study a miniscule “being” with the appropriate tools. In the past, these tools often provided only low-resolution views. A first approach to studying an unknown virus is to know exactly its identity, and to place it into context of other related and non-related viruses. For human and public health, this is important as the identity may provide a course of action to limit the effects of the pathogen.

Virus identification, characterization and taxonomy are necessarily based on the “best-available” methodology and technology. The “best-available” may not be the ideal method. In the past as well as currently, virus attributes used for identification include measurements of the physical viral structure; biological, biochemical and clinical observations; pathogenic properties, antibody recognition of capsid proteins (immunochemistry); and the physical genetic material (genome), including the particular form of genome, genome hybridization and restriction enzyme digestion patterns [2]. Technology is now providing a much better alternative with the development of recombinant DNA protocols and DNA sequencing methods.

The development of DNA sequencing methodologies, particularly the Sanger dideoxynucleotide chain terminator-based chemistry, has provided the ultimate highest resolution data possible: the primary genome sequence. The original validation of this method by sequencing a DNA virus (phi-X174; 5.4 kb) [4] has led to a continuing cycle of data acquisition and analysis in conjunction with technology development, with immediate impacts beyond “mere” viruses. Since 1977, with the tsunami of sequence data, along with rising scientific and public interest, the focus and resources have shifted to the much larger and “sexier” genomes, particularly the human genome and bacterial pathogens. On a smaller scale (seiche) however, viral genomes are being sequenced and studied as well, but with less fanfare. Recently available resources allow for a larger genomic view of viruses, particularly as populations rather than single entities. For example, the National Institute of Allergy and Infectious Disease (NIAID) and the National Institutes of Health (NIH), through the funding of the Genome Sequencing Center (GSC) for Infectious Diseases at the J. Craig Venter Institute (JCVI), have recently approved “white papers” proposing for the large-scale genomic sampling of several groups of viruses: adenovirus, influenza, norovirus, rotavirus, coronavirus, arbovirus and paramyxovirus. Rhinoviruses, as a group, were recently analyzed in such a format allowing for a global examination [5] and more genomes in this family are being sequenced. Development of next generation DNA sequencing technology is providing an additional impetus, given “faster, cheaper” and hopefully higher quality data.

From the first virus genome sequence completed (MS2; 3.6 kb) [6] to that of the recent largest virus (mimivirus; 1.2 Mb) [7], the viral genome and its analysis have revealed high-resolution details of the molecular basis of a particular biological system, along with unexpected and surprising details. As viruses, again, are important health concerns and are also past and current “model organisms”, an “umbrella effect” is taking place, following the collection of genome sequence data. In this scenario, associated viral genome-specific bioinformatic tools are being developed, data sets and databases established, biotechnological tools and applications implemented, and new policies and standards established- all resulting from virus genome sequence data. All of these are presented, as representations, in this special issue, reflecting the continued and growing importance of viral genomics and bioinformatics.

### 1.1. Genomics of Select Virus Families

Although, in general, large data sets of related viral genomes from a family are just being assembled, there are available sets of genomes from members of certain virus families that are of recent and critical interest in the context of human and public health issues. Many of these data sets have either been recently assembled and reported, such as rhinovirus [5], or have been reviewed recently, for example, rhabdoviruses [8], papillomavirus [9] and human immunodeficiency virus (HIV) [10]. The recent publication of these and other similar genomes preclude their inclusion in this special issue.

Rather than attempting to provide a comprehensive survey of many virus families with multiple genomes sequenced and analyzed, a goal of this special issue is to provide a sampling and a range of the virus families that have been and are of importance such that multiple genomes have been sequenced and analyzed using bioinformatic methods. From these contributions, one will see that genomics and bioinformatics are intertwined. At one end of the spectrum are human adenoviruses, which contain DNA genomes of 35 kb. Once considered “large genome” viruses, these are provided in contrast to the much larger genomes found amongst the *Iridoviridae*, with DNA genomes of 106 kb. Eaton *et al.* reviews these. In addition, Hendrickson *et al.* examines the *Poxviridae* genomes, which range from 130 to 375 kb. For comparison, Woo *et al.*, reports on the coronaviruses, with the largest genomes found for RNA viruses (26–38 kb). This report on coronavirus illustrates the importance of viral genomics and bioinformatics in monitoring and identifying global pathogens rapidly and effectively, allowing for measures to be taken. The pathogen responsible for causing a novel highly contagious respiratory disease in Hong Kong (2002–2003), severe acute respiratory syndrome (SARS), was identified as a SARS-related coronavirus by a microarray assay that made use of unique genome signature sequences [11]. A possible origin for this emergent pathogen is presented by Woo *et al.*, using molecular clock analysis to show that the most recent common ancestor of human/civet SARS-related coronovirus appeared in the period between 1999–2002, just preceding the outbreak. They also note recombination between different strains of coronaviruses. In general, genome recombination is a molecular pathway leading to new strains and emergent pathogens, as demonstrated recently in the human adenoviruses [12,13].

Torres *et al.*, in their contribution summarizing the growing number of human adenoviruses genomes, note that there is a “revolution in viral genomics”, exemplified by the bioinformatic analysis of the genomes and an initiative to change the long-standing gold standards for taxonomy and nomenclature. A paradigm change, along with a genomics and bioinformatics-based algorithm, is proposed to use the whole genome attributes, including phylogenomics and compelling changes in the strain’s biology and pathogenicity. This calls for utilizing 100% of the primary sequence data rather than the past use of immunochemical methods, such as serum neutralization, that probed the limited and often murky three-dimensional protein array (epitope) that represents an indirect interpretation of the primary sequence data, the tertiary sequence. As illustrated in a figure by Torres *et al.*, for the human adenoviruses, the hexon-specific loop 1 epitope represents only 2.6% of the genome sequence [13]. Although a genome-based paradigm is surprisingly controversial and has detractors, this approach, along with a genome-based algorithm, has already been implemented in the papillomaviruses (PV) since 2004 [8]. An interesting, but illogical and amusing, comment by detractors of this “revolution in human adenovirus nomenclature” is “there will be too many numbers (new types)”. As noted by Torres *et al.*, there are currently 55 human adenovirus types accepted by peer review; this is less than one-third of the 189 PV types recognized currently [9].

Hendrickson *et al.* provide a contribution examining the *Poxviridae* genomes. Members of this family are highly successful pathogens that infect a range of hosts, and cause may diseases in humans, including smallpox. They are also of interest as naturally occurring pathogens (monkeypox) and potential biothreat agents (smallpox-related). A collection of genomes allows an understanding of the orthologous genes (core genes) shared across this diverse group of viruses. As an example of the differences between genomes of viruses and other organisms and of the ironic complexity of analyzing “simple smaller” viral genomes, a newly developed poxvirus-specific computational tool was used to predict accurate gene sets. This provided a view of reductive evolution, where the reduction in the core gene set, that is, gene loss, plays a proposed critical role in speciation and serves to limit emergent viruses to particular niches. Eaton *et al.*, in their survey of *Iridoviridae*, also examined the concept of core genes within the diverse members of that family. They also note, in addition, that individual genomes contain sets of unique repetitive sequences.

### 1.2. Bioinformatics and Computational Analysis Tools

As noted earlier, viral genomes differ from other organism genomes in complexity, despite their generally smaller sizes and presumed “simplicity”. For example, one problem is that the smaller size of the genome dictates a higher density of gene coding, with all six reading frames utilized. Coding regions frequently overlap. Another problem is that mRNA transcripts often yield multiple spliced products. Some of the exons may be located far apart and may contain very short coding regions. These complicate the analysis of viral genomes and may be barriers to comparative analysis especially if genome annotation standards are not in place. Software tools, such as automated annotation, developed for non-virus genomes may not be transferable directly to the analysis of virus genomes. These issues are critical in virus genomics and bioinformatics. The contributions in this special issue include virus-specific computational tools. Mentioned earlier, Hendrickson *et al.* provide a newly developed poxvirus-specific computational tool for predicting accurate gene sets. Eaton *et al.* noted that individual *Iridoviridae* genomes contain sets of unique repetitive sequences. These “repeats [,] common to more than one virus [,] were also identified and changes in copy number between these repeats may provide a simple method to differentiate between very closely related virus strains”.

Specific software to take advantage of the general nature of unique repetitive sequences in viral genomes was presented in a contribution by Sadeque *et al.* This is an online software tool developed for identifying highly conserved DNA sequences. Again, these conserved sequences may be used to identify specific strains and pathogens, and may be used for tracking a specific strain in outbreaks or for molecular evolution studies. These are also unique genome signatures that can be used as a surveillance or diagnostic tool, such as the ones used to populate an Affymetrix microarray chip developed for respiratory pathogens [14].

Both Eaton *et al.*, examining *Iridoviridae*, and Hendrickson *et al.*, examining *Poxviridae*, note the importance of core gene sets amongst the viruses in their contributions. The wealth of genomes, particularly the bacterial ones originally, has enabled a broader view and analysis of the components that are required, presumably being conserved within a family of genomes [15]. This has led to the concept of a “pan-genome” that comprises a “core” set and a “dispensable” set of genes. The dispensable set presumably allows a unique strain to occupy a specific niche. CoreGenes is an “on-the-fly” web-accessible software tool designed originally (2002) to survey a group of viruses for determining a core set of genes; recently it has been upgraded [16] to provide features desired for the display of an *in silico* proteome used for reclassifying the bacteriophage genomes [17]. Recently another version of this computational tool has been published, noting the division of the pan-genome into a “core” and an “accessory” set of genes. In this definition, the accessory set is believed to be a source of genetic variability allowing for niche adaptations, and is gained through lateral gene transfer [18], which is essentially the definition of the “dispensable” gene set noted above [15]. The notion of a core set of genes being necessary as a minimal set is important in the development of synthetic genomes, a biotechnology application of genome sequence data and bioinformatics.

### 1.3. Biotechnological Applications and Viral Genomics

The application of genomic and bioinformatic methodologies and technologies to virus studies is providing not only a large amount of data, but also is stimulating an increasing number of applications. As mentioned earlier, the genomes (Eaton *et al.*, and Hendrickson *et al.*) and the tools used to mine data from them (Sadeque *et al.*), provide unique sequence signatures that may be used to populate microarray chips for surveillance and diagnostics [11,14] or to develop specific polymerase chain reaction (PCR) assays. A different application is realized in using these approaches to understand in greater detail the viruses that are used in biomedical and biotechnological processes, for example adenoviruses are used in human gene therapy as gene transfer vectors; genomics and bioinformatics allow for a rational approach to designing appropriate and safe vectors [19].

### 1.4. Development of Standards for Viral Genome and Annotation Data

The emerging sets of viral genome data from both virus families and sporadic isolates are prompting a focused interest in establishing standards for consistent and comprehensive genome sequence annotation for viral genomes. The 3rd Prokaryotic Genome Annotation workshop was held on 26–27 April 2010 at the J. Craig Venter Institute (JCVI; Rockville, MD), sponsored by the National Center for Biotechnology Information (NCBI). For the first time, a virus genome Working Group was included, formed and tasked with developing sequence, function, and metadata annotation standards for viral and bacteriophage genomes. In this issue, Brister, *et al.* provide a meeting report to summarize the discussions and issues involved in establishing viral genome annotation standards the NCBI Annotation Workshop. In addition they also review the policy recommendations presented at this meeting. These are necessary, in light of the very large data set of virus genomes that is being generated by many researchers globally. As they noted, there are 27,091 full-length virus genomes deposited in GenBank as of 2010. In the context of the discussion earlier of the on-going recent NIAID-funded projects in the U.S., a significant increase of viral genomes is anticipated in the very near future. Standards and consistencies are vital for the archiving, distribution and use of these data, particularly since international research allows for the deposit of these sequences into one or more public databases: International Nucleotide Sequence Database Collaboration (INSDC), the DNA Database of Japan (DDBJ) [2], the European Nucleotide Archive (ENA) [3], and GenBank [4]. Since the individual data are the responsibility of the researcher, it is imperative for universal standards to be developed and to be imposed for data depositors to avoid “cacophony” and to allow stress-free effective data mining of these databases.

## 2. Summary

Viruses have been known and studied since before the start of the last century. Continued investigations of these “beings” over these many years despite the lack of high-resolution tools reflect the many important roles they play relative to humans. Our understanding of them and our ability to take advantage of them are limited by our lack of complete data, preferably high-resolution, and by the tools that are available. The relevant and important contributions in this special issue, along with the advent of recombinant DNA technology and DNA sequencing methodology near the turn of this century, and with the continuing development of next generation DNA sequencing technology, highlight how genomics and bioinformatics are allowing and will allow a much deeper and broader understanding and appreciation of viruses and bacteriophages. As noted by Torres *et al.*, we are on the cusp of a “revolution in viral genomics”. *Alea iacta est*!

## References

[1] Lechevalier H (1972). Dmitri Iosifovich Ivanovski (1864–1920). Bacteriol Rev.

[2] Flint J, Enquist LW, Racaniello VR, Skalka AM (2008). Principles of Virology.

[3] La Scola B, Desnues C, Pagnier I, Robert C, Barrassi L, Fournous G, Merchat M, Suzan-Monti M, Forterre P, Koonin E, Raoult D (2008). The virophage as a unique parasite of the giant mimivirus. Nature.

[4] Sanger F, Air GM, Barrell BG, Brown NL, Coulson AR, Fiddes CA, Hutchison CA, Slocombe PM, Smith M (1977). Nucleotide sequence of bacteriophage phi X174 DNA. Nature.

[5] Palmenberg AC, Spiro D, Kuzmickas R, Wang S, Djikeng A, Rathe JA, Fraser-Liggett CM, Liggett SB (2009). Sequencing and analyses of all known human rhinovirus genomes reveals structure and evolution. Science.

[6] Fiers W, Contreras R, Duerinck F, Haegeman G, Iserentant D, Merregaert J, Min Jou W, Molemans F, Raeymaekers A, Van den Berghe A, Volckaert G, Ysebaert M (1976). Complete nucleotide sequence of bacteriophage MS2 RNA: primary and secondary structure of replicase gene. Nature.

[7] Le Raoult D, Audic S, Robert C, Abergel C, Renesto P, Ogata H, La Scola B, Suzan M, Claverie J (2004). The 1.2 Megabase genome sequence of mimivirus. Science.

[8] Kuzmin IV, Novella IS, Dietzgen RG, Padhi A, Rupprecht CE (2009). The rhabdoviruses: biodiversity, phylogenetics, and evolution. Infect Genet Evol.

[9] Bernard HU, Burk RD, Chen Z, van Doorslaer K, Hausen H, de Villiers EM (2010). Classification of papillomaviruses (PVs) based on 189 PV types and proposal of taxonomic amendments. Virology.

[10] Los Alamos National Laboratory (LANL), HIV Sequence Compendium 2010. http://www.hiv.lanl.gov/content/sequence/HIV/COMPENDIUM/2010/sequence2010.pdf.

[11] Rota PA, Oberste MS, Monroe SS, Nix WA, Campagnoli R, Icenogle JP, Peñaranda S, Bankamp B, Maher K, Chen MH, Tong S, Tamin A, Lowe L, Frace M, DeRisi JL, Chen Q, Wang D, Erdman DD, Peret TC, Burns C, Ksiazek TG, Rollin PE, Sanchez A, Liffick S, Holloway B, Limor J, McCaustland K, Olsen-Rasmussen M, Fouchier R, Günther S, Osterhaus AD, Drosten C, Pallansch MA, Anderson LJ, Bellini WJ (2003). Characterization of a novel coronavirus associated with severe acute respiratory syndrome. Science.

[12] Walsh MP, Chintakuntlawar A, Robinson CM, Madisch I, Harrach B, Hudson NR, Schnurr D, Heim A, Chodosh J, Seto D, Jones MS (2009). Evidence of molecular evolution driven by recombination events influencing tropism in a novel human adenovirus that causes epidemic keratoconjunctivitis. PLoS One.

[13] Walsh MP, Seto J, Jones MS, Chodosh J, Xu W, Seto D (2010). Computational analysis identifies human adenovirus type 55 as a re-emergent acute respiratory disease pathogen. J Clin Microbiol.

[14] Lin B, Wang Z, Vora GJ, Thornton JA, Schnur JM, Thach DC, Blaney KM, Ligler AG, Malanoski AP, Santiago J, Walter EA, Agan BK, Metzgar D, Seto D, Daum LT, Kruzelock R, Rowley RK, Hanson EH, Tibbetts C, Stenger DA (2006). Broad-spectrum respiratory tract pathogen identification using resequencing DNA microarrays. Genome Res.

[15] Medini D, Donati C, Tettelin H, Masignani V, Rappuoli R (2005). The microbial pan-genome. Curr Opin Genet Dev.

[16] Mahadevan P, King JF, Seto D (2009). CGUG: *in silico* proteome and genome parsing tool for the determination of “core” and unique genes in the analysis of genomes up to *ca.* 1.9 Mb. BMC Res Notes.

[17] Lavigne R, Darius P, Summer EJ, Seto D, Mahadevan P, Nilsson AS, Ackermann HW, Kropinski AM (2009). Classification of Myoviridae bacteriophages using protein sequence similarity. BMC Microbiol.

[18] Laing C, Buchanan C, Taboada EN, Zhang Y, Kropinski A, Villegas A, Thomas JE, Gannon VP (2010). Pan-genome sequence analysis using Panseq: an online tool for the rapid analysis of core and accessory genomic regions. BMC Bioinformatics.

[19] Seto J, Walsh MP, Mahadevan P, Zhang Q, Seto D (2010). Applying genomic and bionformatic resources to human adenovirus genomes for use in vaccine development and for applications in vector development for gene delivery. Viruses.

